# Beyond the Heart‐Lung Axis: A Review of the Crosstalk Between the Hemodynamic, Neurohormonal, and Immune Systems in Pulmonary Hypertension

**DOI:** 10.1002/cph4.70266

**Published:** 2026-09-22

**Authors:** Alejandro A. Vega, Juliet M. Alfaro, David S. Pinto, Maryam Emamimeybodi, Soban Umar

**Affiliations:** ^1^ Department of Anesthesiology and Perioperative Medicine University of California, Los Angeles Los Angeles California USA; ^2^ Division of Vascular Surgery, Department of Surgery University of California, Los Angeles Los Angeles California USA; ^3^ David Geffen School of Medicine University of California, Los Angeles Los Angeles California USA; ^4^ Department of Integrative Biology and Physiology University of California, Los Angeles Los Angeles California USA; ^5^ Molecular, Cellular, and Integrative Physiology Graduate Program University of California, Los Angeles Los Angeles California USA; ^6^ Department of Internal Medicine UC Davis Health Sacramento California USA

## Abstract

Investigating systemic interactions involving interorgan crosstalk in pulmonary arterial hypertension (PAH) may elucidate the underlying mechanisms that drive pathogenic processes. Further understanding of the disease process through communication pathways at the broader physiological level can inform advancements in novel therapeutic approaches. This article is a comprehensive summary of our current knowledge surrounding the heart‐lung axis in PAH as well as systemic pathophysiological contributions and their methods of communication through neurohormonal, inflammatory, metabolic, and extracellular mediated pathways. Neurohormonal crosstalk encompasses the renin‐angiotensin‐aldosterone system (RAAS) and sympathetic nervous system (SNS), both of which modulate communication between the heart and lung. Inflammation, within the heart‐lung axis and at the systemic level, is characterized by cytokine signaling and chemokine recruitment. Extrapulmonary mediators of inflammation in PAH include the liver, chronic kidney disease (CKD), adipose tissue, insulin resistance, and bone marrow‐derived cells. Extracellular vesicles mediate proximal to distal communication pathways, spanning from localized crosstalk between the heart and lungs to peripheral interorgan signaling. Emerging studies have revealed broader physiological contributions across multiple organ systems in PAH pathogenesis, such as pulmonary vascular remodeling and endothelial cell dysfunction, suggesting systemic interorgan crosstalk with the heart and lungs influences the disease phenotype.

## Graphical abstract

Pulmonary hypertension is driven by dynamic crosstalk beyond the physical connection between the heart and lung, involving neurohormonal systems, immune pathways, and peripheral organs. This review highlights how multisystem signaling contributes to disease progression and may reveal new diagnostic and therapeutic targets.

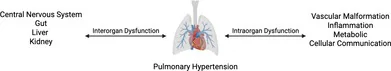

## 1 Introduction

The cardiopulmonary unit functions as an intertwined system composed of the heart, lungs and the blood vessels connecting them. Traditionally, the relationship between these components has been described in purely mechanical terms, focusing on measurements, such as pressure, resistance, ventricular‐arterial coupling, compliance and more as the main determinants of function and clinical trajectory. Under normal conditions, the pulmonary circuit is a low‐resistance, high‐compliance vascular bed (Konstam et al. 2018; Harrison et al. 2015). However, in some diseased states, the vasculature is affected, and medial hypertrophy occurs, along with adventitial fibrosis, which contributes to elevated pulmonary vascular resistance (PVR) and decreased compliance of the blood vessels. These changes impose an increased load on the right ventricle (RV), which may result in maladaptive remodeling such as compensatory hypertrophy, RV dilation, and ultimately right‐sided heart failure. Right‐sided failure is still one of the leading causes of mortality for several forms of pulmonary vascular disease (PVD) despite our increased understanding and advances in therapy (Ruopp and Cockrill 2022; Veerdonk et al. 2011).

Among pulmonary vascular diseases, pulmonary artery hypertension (PAH) stands as a prime example of the pathophysiology mentioned above, ultimately leading to death if untreated. In the 1990's, the median survival rate in patients with pulmonary hypertension was 2.8 years, with single year survival rates at 1 year (68%), 3 years (48%), and 5 years (34%) (D'Alonzo et al. 1991). However, due to developments in our understanding of pulmonary hypertension and the development of therapies targeting key biological pathways such as the prostacyclin pathway and endothelin pathway, combination therapies have increased the five‐year survival rate to 60% in 2015 (Ruopp and Cockrill 2022; Masih et al. 2025).

Beyond the mechanical measurements mentioned earlier, there has been accumulating evidence indicating that the RV, lungs, and pulmonary vasculature communicate via indirect bidirectional pathways. Some of these additional methods of communication include interacting via neurohormonal, inflammatory, metabolic, and extracellular vesicle‐mediated pathways. These pathways indicate that PVD is not a disease simply of elevated vascular resistance but rather a whole‐body disorder of heart and lung crosstalk via several pathways. This review aims to move beyond the typical hemodynamic framework and instead present pulmonary vascular disease as a disorder of integrated, multi‐organ cardiopulmonary communication, while highlighting emerging opportunities to translate these insights into more comprehensive diagnostic and therapeutic strategies.

## 2 Neurohormonal Crosstalk

Previously, the activation of several neurohormonal systems such as the renin‐angiotensin‐aldosterone system (RAAS), sympathetic nervous system (SNS), and natriuretic peptide (NP) pathways was thought to be secondary to hemodynamic stress. However, recent studies suggest that these systems participate in bidirectional communication between the pulmonary vasculature and the RV and are a major mediator of heart and lung communication. Having a better understanding of these system interactions has allowed us to view these systems as a dynamic conduit through which the heart and lungs influence one another as opposed to background noise in response to hemodynamic stress.

### 2.1 Renin‐Angiotensin‐Aldosterone System (RAAS)

The Angiotensin‐Converting Enzyme (ACE)/Angiotensin II (Ang II)/Angiotensin II Type 1 Receptor (AT1R) axis, also known as the ACE/Ang II/AT1R axis, is what is normally thought of as the classical and dominant portion of the RAAS system. During this axis, ACE converts Angiotensin I into Angiotensin II, which then binds to the AT1R receptor. Under normal conditions, this axis is responsible for regulating blood pressure and fluid balance, but when overactivated, it can lead to cardiovascular damage by promoting aberrant proliferation, limiting apoptosis, inducing vasoconstriction, hypertrophy, inflammation, and fibrosis (de Man et al. 2012; Mehta and Griendling 2007; Morrell et al. 1997; Papavassiliou et al. 2023). The vascular changes can result in increased pulmonary vascular resistance and lower the compliance of the arterial pulmonary vasculature, resulting in an increased afterload on the RV, which then triggers the RAAS cascade, ultimately resulting in a detrimental feedback loop (Humbert et al. 2022). Emerging research trends have resulted in a bigger focus on the ACE2/Ang‐(107)/Mas‐r axis, where angiotensin‐converting enzyme 2 (ACE2) produces the heptapeptide Angiotensin‐(1–7) (Ang‐(1–7)), which binds to the Mas receptor (Mas‐R). This axis can be thought of as the counter‐axis to the ACE/Ang II/AT1R axis. The ACE2/Ang‐(107)/Mas‐r axis is essentially a protective component of the RAAS system that counteracts the detrimental effects of the classical RAAS pathway that was discussed previously (Bader et al. 2024). It does so by increasing nitric oxide levels to cause vasodilation, minimize inflammation, improve cell metabolism, and decrease fibrosis (Papavassiliou et al. 2023; Liu et al. 2018; Lang et al. 2025). Rat studies have shown that ACE2 activation with diminazene, when combined with vascular endothelial growth factor (VEGF) inhibitors, may prevent and attenuate the development of PAH in animals and thus may be a potential therapeutic strategy for the treatment of PAH in humans (Chen et al. 2021).

### 2.2 Sympathetic Nervous System (SNS) Overactivation

Patients with PAH have been shown to consistently have a higher level of sympathetic activity compared to healthy individuals. Using direct microneurography, researchers showed elevated muscle sympathetic nerve activity (MSNA) in individuals with PAH (Velez‐Roa et al. 2004). This increase in MSNA has additionally been linked to worse clinical outcomes and higher pulmonary pressure, declining functional class, and reduced survivability (Ciarka et al. 2010). Some studies show that hyperoxia resulted in a suppressed MSNA, indicating that chemoreflex activation due to low O_2_ levels may serve as a driver of sympathetic overactivity (Velez‐Roa et al. 2004). From a mechanistic point of view, hypoxemia and pulmonary arterial stiffening, both characteristics of many diseased lung states, result in a reflex arc. Traditionally, the peripheral chemoreceptors in the carotid body and aortic arch can detect changes in O_2_, CO_2_, and pH (Iturriaga et al. 2021). When these receptors sense the low oxygen state, Type 1 “glomus” cells within the carotid body become depolarized, triggering the release of neurotransmitters, ultimately exciting the afferent nerve fibers of the glossopharyngeal nerve (Cranial Nerve IX). This signal is then sent to the medullary cardiorespiratory centers in the brainstem, increasing the sympathetic signal resulting in an increased rate of ventilation (Iturriaga et al. 2021; Prabhakar and Semenza 2015). Additionally, baroreceptors in the carotid sinuses and aortic arch undergo baroreceptor unloading due to stiffened arteries, thus dampening the rate of neuronal firing. This reduction in the firing rate results in an increased sympathetic drive. Together, these two pathways act on the central autonomic network, resulting in an increased efferent sympathetic tone. This increased tone results in pulmonary vasoconstriction, increasing pulmonary vascular resistance and RV afterload (Sun and Chan 2018; Vaillancourt et al. 2017). Initially, this increased sympathetic tone results in increased levels of catecholamines, which in the short term helps the RV overcome the increased afterload by having it contract more forcibly. In the long term, these elevated catecholamines lead to β1‐adrenergic receptor desensitization and downregulation (Ryan and Archer 2014; Maron and Leopold 2015). β2 adrenergic receptors, which are normally in caveolae, can undergo maladaptive remodeling that can occur under chronic stimulation, resulting in maladaptive pathways (Sun et al. 2018; Macdougall et al. 2012). Combined, these two receptor changes will result in high adrenergic drive but a low adrenergic response. These adrenergic changes show us that pulmonary vascular pathology goes well beyond the physical connection between the heart and lungs. These two organs communicate via neural reflex arcs that amplify sympathetic flow in certain diseased states.

These mechanistic insights have potentially important therapeutic implications, as interventional strategies targeting autonomic regulation are actively being explored. Some of these strategies include pulmonary artery denervation (PADN) and vagus nerve stimulation, both of which aim to help rebalance sympathetic and parasympathetic signaling and may improve pulmonary vascular tone and right ventricular function.

### 2.3 Natriuretic Peptides

As mentioned in the earlier section, the activation of the RAAS system can result in changes in fluid status, along with blood pressure and salts. When the volume status increases, the natriuretic peptide (NP) system acts as one of the most important counter‐regulatory pathways to the RAAS system and as a result serves as a link between the RV and pulmonary vasculature. This system acts as a protective signal from the RV and atria to try and prevent maladaptive remodeling (Yap et al. 2004; Kuwahara 2021).

In states of volume overload, mechanical stretch of the right atria (RA) and RV stimulates the release of atrial natriuretic peptide (ANP) and B‐type natriuretic peptide (BNP) (Kuwahara 2021). They then bind primarily to natriuretic peptide receptor‐A (NPR‐A/GC‐A) on vascular smooth muscle and endothelial cells, resulting in the activation of cGMP protein kinase G pathways, which lead to vasodilation, limited smooth muscle proliferation, and antifibrotic signaling (Sangaralingham et al. 2022). From a clinical point of view, the circulating levels of BNP and NT‐proBNP, both derived from proBNP, can serve as robust markers of RV strain (Maurer et al. 2023). Elevated levels have been shown to be predictors of severe disease and, as such, have become part of several risk stratification frameworks, including REVEAL and COMPERA, as well as major guideline‐based assessments such as ACC/AHA/HFSA heart failure guidelines and ESC/ERS pulmonary hypertension guidelines (Humbert et al. 2022; Benza et al. 2019; Hoeper et al. 2022; Heidenreich et al. 2022). Ultimately, elevated levels correlate with impaired exercise capacity, increased RV strain, worse hemodynamics, and increased mortality (Maurer et al. 2023; Lewis et al. 2020; Chang et al. 2021).

In recent years, studies have advanced our understanding of the pulmonary vasculature as a regulator of NP signaling and not simply a physical connection from the heart to lungs (Dignam, Aubdool, et al. 2025). NPR‐C receptors which are expressed in several tissues, and are highly expressed on pulmonary endothelial cells, act as clearance receptors by binding ANP, BNP and C‐type natriuretic peptide (CNP) from circulation. In healthy individuals, this maintains balance by controlling levels of NPs available to elicit downstream effects. However, in pathological conditions, excessive NPR‐C activity may be observed, resulting in excess NPs being removed from circulation, and as a result, removing or minimizing the protective effect of NPs. In recent years, the understanding of NPR‐C has been expanded, and instead of solely acting as a clearance sink, some pre‐clinical studies suggest that targeting these specific natriuretic peptides may be a possible pharmacological target for treating hypertension. In one study, it was found that pharmacologic modulation of the NPR‐C via a specific natriuretic peptides clearance receptors' agonist, cANF4‐23 (cANF), lowered pulmonary artery pressures in mice and improved RV function (Egom et al. 2017). Additionally, in the same study, it was found that mice lacking NPR‐C were more susceptible to developing HF when compared to their counterparts, indicating NPR‐C may play a protective role in the heart.

Another novel area of research is the study of C‐type natriuretic peptide (CNP). CNP has long been considered to be an endothelial paracrine factor; however, it is now also being recognized as a regulator of pulmonary vascular remodeling. Specifically, the CNP/GC‐B/cMP pathway is a signaling pathway that becomes activated when the peptide CNP binds to its receptor, a Guanylyl cyclase‐B (GC‐B), which leads to the production of the secondary messenger cGMP (commonly known as cyclic guanosine monophosphate). This secondary messenger then activates downstream proteins that regulate cell growth, vascular tone, and differentiation. In one study, PAH pericytes were shown to have preserved CNP/GC‐B/cMP signaling, which resulted in the suppression of proliferation, migration, and mesenchymal transdifferentiation (Dabral et al. 2024). It is believed CNP attenuates the changes that are seen during PAH, which includes enhanced glycolysis, glucose uptake, and nucleotide biosynthesis (Noh et al. 2025). Additionally, the capacity of CNP to reduce RV afterload and limit adverse remodeling in PH indicates that CNP may play a role as a local pulmonary vascular signal that contributes to maintaining vascular homeostasis and right ventricular function (Dignam, Aubdool, et al. 2025). Ultimately, these three peptides mentioned form a multidirectional signaling network between the myocardium and pulmonary vasculature.

In recent years, strategies focusing on the enhancement of natriuretic peptide signaling have moved from concepts towards clinical practice. For example, some researchers have blocked neprilysin‐mediated NP degradation with the use of angiotensin receptor blocker and neprilysin inhibitor, sacubitril/valsartan (Sac/Val, LCZ696), resulting in the increased peptide bioavailability. Their findings showed a reduction in pulmonary pressures, vascular remodeling, and RV hypertrophy in a rat model of PH, indicating that the blocking of neprilysin‐mediated NP degradation may serve as a possible treatment of PH and RV dysfunction (Clements et al. 2019). Multiple preclinical rodent models of PH and RV pressure overload have demonstrated that sacubitril/valsartan administration results in the lowering of the pulmonary and right ventricular systolic pressure, resulting in the attenuation of RV hypertrophy and remodeling (Liu et al. 2021; Sharifi Kia et al. 2020). Furthermore, in some studies looking at patients with varying types of heart failure complicated by pulmonary hypertension, there have been reports of a decrease in pulmonary pressures and improvement in the function of the RV after Angiotensin Receptor‐Neprilysin Inhibitor (ARNI) therapy (Xu et al. 2023; Zhang et al. 2022). However, it is important to note that to properly determine the true impact ARNI may have on PAH, randomized trials are still needed.

## 3 Inflammatory and Immune Mediators

Inflammation is now recognized as one of the ways in which bidirectional signals are sent between the heart, lungs, and rest of the body, which contributes to the development and progression of pulmonary vascular disease (PVD) and right‐ventricular dysfunction. It is ultimately the combination of cytokines, chemokines, and immune cell migration that contribute significantly to vascular and myocardial remodeling. It is important to note that there are many other compounds besides the ones listed above that play a role in the interplay between the heart and lung, but for the purposes of this review, we will focus on the most central components.

### 3.1 Cytokine Signaling

Although there are several cytokines that contribute to the intercommunication between the heart and lungs in PH, the most central cytokines include interleukin‐6 (IL‐6), tumor necrosis factor α (TNF‐α), and interleukin‐1β (IL‐1β). Studies show that elevated levels of IL‐6 are correlated with pulmonary vascular resistance, functional class, and mortality in PAH (Prins et al. 2018; Selimovic et al. 2009). These downstream effects are likely due to IL‐6 promoting smooth muscle proliferation using the STAT3 pathway, ultimately resulting in endothelial‐to‐mesenchymal transitioning (EndMT), which ultimately contributes to the narrowing of the lumen (Fu et al. 2021; Cai et al. 2018). However, in the RV, IL‐6 signaling results in the activation of the gp130/STAT3 pathway, which initially contributes to the development of adaptive hypertrophy. However, over time, it leads to maladaptive fibrosis and contractile dysfunction (Fischer and Hilfiker‐Kleiner 2007).

TNF‐α has been shown to impair RV contractility by altering calcium handling, which results in the induction of cardiomyocyte apoptosis through NF‐κB dependent pathways (Hamid et al. 2009). In the pulmonary vasculature, it upregulates adhesion molecules, such as VCAM01 and ICAM‐1, which facilitate the infiltration of leukocytes, resulting in increased perivascular inflammation (De Caterina et al. 1995). Furthermore, this effect is compounded by the effects of IL‐1β, which stimulates fibroblast activation and matrix remodeling (Smolgovsky et al. 2021). Together, these cytokines, chemokines and immune cells show that there are mediators that originate from the lung or heart and ultimately propagate, contributing to maladaptive remodeling in both of these organs. One such potential therapeutic target includes IL‐1 receptor antagonist. A preclinical study in rodent models showed that blockage of IL‐1 with anakinra, a known IL‐1 receptor antagonist, resulted in the blunting of pulmonary vascular remodeling and improved RV function (Voelkel et al. 1994). Furthermore, in a single‐arm, open‐label phase 1B/II trial, IL‐1 blockade was associated with a reduction in systemic inflammatory markers and improvements in right ventricular function (Trankle et al. 2019). Together, these findings support continued investigation of anakinra as a therapeutic approach to limit inflammation in PAH and RV failure.

In addition to right ventricle‐pulmonary vascular interactions, emerging evidence suggests that right ventricle‐left ventricle (RV‐LV) coupling contributes to disease progression in pulmonary arterial hypertension. Progressive RV pressure overload and dilation can shift the interventricular septum towards the left ventricle, resulting in impaired diastolic filling and reduced cardiac output. Beyond these mechanical effects, shared and inflammatory signaling pathways, including sympathetic activation and circulating cytokines, may propagate maladaptive remodeling in both ventricles, establishing a biventricular loop that further amplifies disease progression.

### 3.2 Chemokines and the Recruitment of Immune Cells

Like many other parts of our body, the use of chemokines can be used to attract and accumulate the number of immune cells, including macrophages, T cells, and dendritic cells. These cells play an important role and can accumulate in both the pulmonary artery walls and RV myocardium (Hu et al. 2020; Funes et al. 2018). Several studies have shown that vascular remodeling occurs in both experimental and human PAH tissues and that these tissues have increased expression of CCL2 (MCP‐1) and CXCL10 (IP‐10), which are believed to facilitate both monocyte and macrophage infiltration in these tissues, ultimately contributing to vascular remodeling (Cunningham et al. 2022; Stenmark et al. 2013; Stacher et al. 2012). Other studies find similar chemokine signatures in the RV, which may suggest that the recruitment of the cells mentioned previously may occur simultaneously in both areas (Waehre et al. 2012; Zagorski et al. 2008).

### 3.3 Shared Inflammatory Communication Between the Lungs and RV

Emerging data suggests that the RV and pulmonary vasculature do not operate as discrete isolated units, but rather as two components of a shared inflammatory axis. In pulmonary vascular disease, injured pulmonary endothelial cells can release extracellular vesicles (EVs), which can contain inflammatory mediators and microRNA, which can disseminate inflammatory and remodeling signals well beyond their source of origin (Letsiou and Bauer 2018). In the RV, stressors such as pressure overload have been associated with local inflammation, metabolic and mitochondrial dysfunction, and altered gene expression in the RV (Hwang et al. 2021; Qin et al. 2022). Some experimental studies show that HMGB1, a known damage‐associated molecular pattern (DAMP), can be released and enter pulmonary circulation, thus exacerbating vascular inflammation (Zemskova et al. 2020; Dai et al. 2019). Thus, as a result of these modes of communication, it can be seen that the RV and lungs communicate bidirectionally with one another.

Additionally, growing evidence shows that DAMP‐mediated signaling may also further amplify ongoing inflammation through activation of the NLRP3 inflammasome. This process occurs in two steps, which is initiated by either a pattern‐recognition receptor or inflammatory cytokine receptor activating NF‐κB, thus increasing expression of NLRP3 and pro‐IL‐1β which primes the inflammasome (Bauernfeind et al. 2009). Secondly, a subsequent activation signal, such as extracellular ATP‐P2X7 signaling, potassium efflux, or mitochondrial dysfunction resulting in increased reactive oxygen species, ultimately in conjunction, promotes assembly of the NLRP3‐ASC‐procaspace‐1 complex (Jo et al. 2016; Kelley et al. 2019). These caspases then cleave pro‐IL‐1β and pro‐IL‐18 into their IL‐1β and IL‐18 mature forms, respectively. Several studies have implicated this pathway in vascular inflammation, endothelial injury and dysfunction, adverse right‐ventricular remodeling and pulmonary vascular remodeling, all of which are important components of disease pathogenesis in pulmonary hypertension (Mavrogiannis et al. 2022; Al‐Qazazi et al. 2022). Therefore, the NLRP3 axis provides a link between tissue injury and the subsequent DAMP release, innate immune activation, and broader inflammatory signals that are believed to be involved in pulmonary vascular disease.

### 3.4 Therapeutic Strategies Based on Inflammatory Signaling

Due to our increased understanding there is now more research attempting to further categorize this inflammatory crosstalk. One pathway of interest is the Interleukin‐6/interleukin‐21 signaling axis, which has been shown to be critical for the development of PAH in rodent models (Hashimoto‐Kataoka et al. 2015). As a result, some researchers have attempted to target this axis. In one PAH trial, Tocilizumab, an IL‐6 receptor antagonist, showed a good safety profile in PAH trials but did not result in significant improvement in hemodynamic measurements (Toshner et al. 2022). Additionally, in other studies Anakinra (IL‐1 receptor blocker) and Etanercept (TNF‐α inhibitor) have shown preclinical efficacy in not only reversing pulmonary vascular remodeling but improving RV hemodynamics as well (Hurst et al. 2017; Zhang et al. 2016). In recent years, the use and study of sodium‐glucose co‐transporter‐2 inhibitors and GLP‐1 receptor agonists has increased, and it is found that they may lower systemic inflammation and exert possible secondary benefits on the heart‐lung system (Preda et al. 2024). One preclinical study found that the SGLT2 inhibitor, Empagliflozin, attenuates pulmonary arterial remodeling through the activation of the Peroxisome Proliferator‐Activated Receptor (PPAR) Gamma, indicating that SGLT2 may have a protective role in PAH for humans, but further studies are needed (Lai et al. 2024). Additionally, there has been more interest in targeting cell trafficking pathways, such as using CCR2 and CCR5 antagonism to block monocyte recruitment and CXCR3 to limit infiltration of T‐cells, which are thought to play a key role in the pathogenesis of PAH (Tsuboya et al. 2025).

## 4 Extracellular Vesicles and Exosomes

Extracellular vesicles are a broad category of vesicles that include exosomes, microvesicles, and apoptotic bodies that in recent years have emerged as one of the ways the lungs and RV communicate with one another. These vesicles or transporters can transport microRNA, proteins, lipids, and metabolites that can alter the behavior of the corresponding recipient cells. It's important to note that the composition and contents of the vesicles reflect the vesicles' origin (Benza et al. 2019; Hoeper et al. 2022). In PAH, endothelial cells release exosomes rich in microRNA such as miR‐21, miR‐130/301, miR‐210, and miR‐143/145 (Ranchoux et al. 2018; Deng et al. 2015). All of these microRNAs have been associated with smooth muscle proliferation, endothelial injury, and metabolic reprogramming. One of the most prominent examples includes the release of vesicles containing miR‐210 from endothelial cells under hypoxic conditions, which ultimately results in the suppression of mitochondrial respiration in target cells (Guan et al. 2019).

Proteomic studies looking at patients with PAH have shown to have increased levels of transforming growth factor beta‐associated proteins, matrix degradation products, inflammatory mediators, and endothelin pathway components (Qin et al. 2021; Tomaszewski et al. 2021). When looking at the metabolic profiles of these vesicles they show signatures that ultimately reflect the metabolic dysfunction characteristics of pulmonary hypertension, which as discussed previously, include augmented glycolysis, reduced oxidative phosphorylation intermediates and dysregulation of amino acid metabolism. This demonstrates that extracellular vesicles preserve information about the metabolic and signaling status of the pulmonary vasculature.

### 4.1 Communication Between the Lung and Heart

Evidence throughout the years suggests that extracellular vesicles released from the endothelium of injured lungs can exert direct effects on the RV (Aliotta et al. 2016). This is due to these vesicles affecting the handling of calcium, reducing mitochondrial function, and activating hypertrophic pathways in the RV (Corssac et al. 2021). As mentioned, some of these microRNA include miR‐21 and miR‐210, which downregulate mitochondrial regulation proteins, resulting in limited or reduced oxidative phosphorylation within the affected cardiomyocytes. Other exosomes induce a proinflammatory state and activate fibrotic proteins, thus further impacting the RV (Aliotta et al. 2016). Ultimately, this shows that the pulmonary vasculature has an influence on the RV through the secretion of vesicles that alter the RV biology at a molecular level.

On the other end of the spectrum, an RV that is failing or pressure overloaded releases vesicles that can affect the pulmonary circulation. They do so by releasing compounds which alter the integrity of the endothelium, thus increasing permeability and promoting inflammation. Some of these vesicles can contain miR‐208, miR‐24a, and pro apoptotic proteins that impair the endothelium's ability to repair itself and thus they contribute to vascular remodeling (Carter et al. 2022; Gartz and Strande 2018).

## 5 Interorgan Crosstalk Involving Other Organs in PAH

**FIGURE 1:**
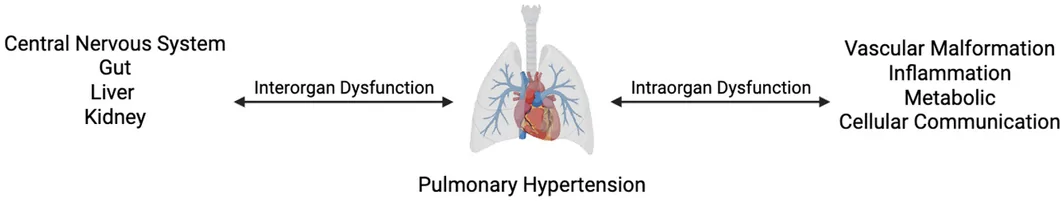
Hypothetical illustration of interorgan crosstalk in pulmonary hypertension (PH). The disease process in PH can be characterized as a systemic disorder, constituting broader physiological interactions throughout the body at the interorgan and intraorgan levels. Neural circuits in the brainstem and spinal cord are characterized by autonomic dysregulation in PH, where afferent signals from cardiopulmonary neurons perpetuate disease progression through neuroinflammation and efferent sympathoexcitation. Elevated systemic venous pressure and vascular remodeling stimulated by PH increased retinal vascular tortuosity. Altered pulmonary vasculature stems from hematopoietic stem cells signaling cytokine upregulation in the lung, while extracellular vesicles released by pulmonary vasculature induce pathogenic endothelial progenitor cell differentiation in the bone marrow. Insulin resistance stimulates upregulation of obesity‐related inflammatory cytokines, perpetuating the effects of systemic inflammation. Right ventricular failure and decreased cardiac output trigger overactivation of the renin‐angiotensin‐aldosterone system (RAAS), leading to progressive kidney failure, consequently impacting pulmonary vascular remodeling through activation of inflammatory pathways and enhanced oxidative stress due to an excess of reactive oxygen species (ROS). In response to systemic inflammation, the liver upregulates proinflammatory pathways to increase leukocyte recruitment to pulmonary endothelial cells, thereby promoting their dysfunction. In turn, upregulation of proinflammatory signaling and PH pathogenesis coincides with the onset of hepatic fibrosis. The hypoxic pathogenic state coupled with systemic inflammation in PH promotes mitochondrial dysfunction and fiber atrophy in skeletal muscle, resulting in reduced exercise capacity. Gut dysbiosis in the gut microbiome is driven by PH pathogenesis, consequently provoking pro‐inflammatory endotoxin secretion.

**FIGURE 2:**
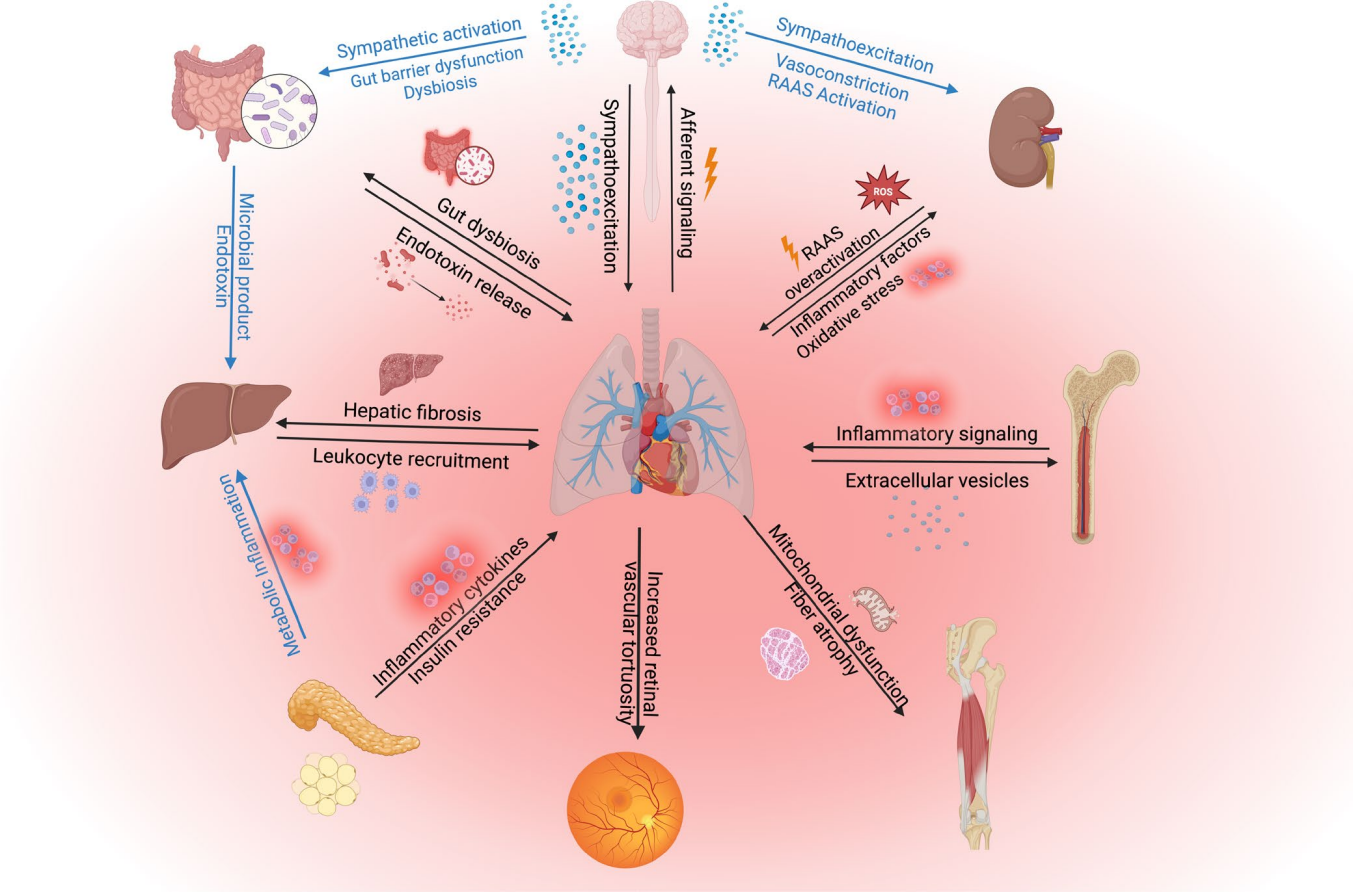
Multisystem crosstalk in pulmonary hypertension. Schematic depicting interactions between the cardiopulmonary unit and peripheral organs through neurohormonal, inflammatory, metabolic, oxidative stress, and extracellular vesicle mediated pathways.

Beyond the heart and lungs, there is accruing evidence indicating that pulmonary vascular disease involves many other organ systems outside those mentioned in this review. Central neural circuits in the brainstem and spinal cord have been shown to modulate sympathetic outflow as a response to pulmonary hypoxia and vascular stiffness, which in turn influences systemic vascular tone and thus contributes to autonomic imbalance. Skeletal muscles exhibit mitochondrial dysfunction, fiber atrophy, and impaired oxygen utilization, all of which have been correlated to reduce exercise capacity. Additionally, the gastrointestinal system may be affected by congestion‐related enteropathy, altered nutrient absorption, and gut microbiome changes that may contribute to inflammation (Figure 1). These systemic effects may be interconnected through metabolic and neurohumoral signaling. Metabolic endotoxemia and adipose‐derived cytokines, including IL‐6 and TNF‐α, link gut dysbiosis and obesity to inflammation and metabolic dysfunction in the liver, adipose tissue, and pancreas, potentially promoting pulmonary vascular remodeling (Dignam, Sharma, et al. 2025; Irwin et al. 2014; Cani et al. 2007). In parallel, altered hypothalamic signaling and sympathoexcitation are associated with intestinal barrier dysfunction and dysbiosis, whose microbial and inflammatory signals may further reinforce neuroinflammation (de Man et al. 2012; Velez‐Roa et al. 2004; Ciarka et al. 2010; Hu et al. 2015). Central and renal sympathetic activation also stimulates vasoconstriction and the renin–angiotensin–aldosterone system, promoting sodium and fluid retention, oxidative stress, and pulmonary vascular remodeling in PAH (Sharma, Oliveira, Yang, Kim, et al. 2020; Sharma, Oliveira, Yang, Karas, et al. 2020) (Figure 2).

Emerging studies are beginning to demonstrate the liver is an essential mediator of inflammatory and angiocrine signaling that modulates pulmonary endothelial cell dysfunction. The proposed mechanism is that the liver reflects immune activation through upregulation of pro‐inflammatory signaling pathways, such as TGFβ and IL‐6 signaling, and implements distal effects on pulmonary endothelial cells through enrichment of inflammatory gene pathways, such as VCAM1, to stimulate recruitment of leukocytes within the pulmonary vasculature (Singh, Lawson, et al. 2025; Singh, Volpicelli, et al. 2025). This elevation of inflammatory signaling also corresponds with increased hepatic fibrosis (Singh, Lawson, et al. 2025). As reflected in Figure 1, in response to elevated inflammatory pathways, hepatic growth factor signaling is characterized in a paradoxical manner, where deficiency in PH animal models and elevation in human plasma analogously amplify liver fibrosis (Singh, Volpicelli, et al. 2025). Circulating hepatic factors secreted by the liver are crucial in maintaining the structural integrity of pulmonary vasculature, and in their absence aberrant angiogenesis compromises vascular tone, leading to the development of pulmonary arteriovenous malformations (PAVMs) (González‐Teshima et al. 2025). As observed in patients with Fontan circulation, PAVMs impose pulmonary limitations through progressive hypoxia, driving pulmonary vascular resistance, subsequently decreasing cardiac output and consequently promoting Fontan circulatory failure (Abdulkarim et al. 2023) While further investigation is needed, recent findings indicate hepatic contributions perpetuate systemic inflammation, subsequently promoting pulmonary endothelial dysregulation.

Chronic kidney disease (CKD) is often associated with connective tissue disorders as well as liver disorders, and these pathologies are also suggested to be key contributors to the onset of pre‐capillary PH (Bolignano et al. 2018). CKD has been implicated in promoting endothelial dysfunction and pulmonary vascular remodeling, thus perpetuating the emergence of PH in these patients. As a hallmark of PH pathogenesis and prominent component of CKD, endothelial dysfunction is amplified through depletion of nitric oxide synthesis, which is consequently associated with risk factors such as diabetes and obesity (Yuan et al. 2024; Tuttle et al. 2024). Uremia induced oxidative stress, upregulation of pro‐inflammatory factors, hypoxemia, and mineral metabolism disorders all contribute to pulmonary vascular remodeling (Yuan et al. 2024). Conversely, PH has been implicated in triggering the pathogenesis of right‐heart cardiorenal syndrome in CKD (Figure 1). Concurrent right ventricular failure and decreased cardiac output in PH results in elevated renal venous congestion and overactivation of the renin‐angiotensin‐aldosterone system, subsequently resulting in salt avidity and hypervolemia (Zeder et al. 2024). In turn, this leads to progressive kidney failure, which impacts pulmonary arterial remodeling through activation of downstream pro‐inflammatory pathways (Zeder et al. 2024; Ebrahimi et al. 2026).

The involvement of obesity and adipose tissue in PH pathogenesis remains widely unknown. Inconsistencies surrounding obesity and its role in PH constitute the “obesity paradox,” where, counterintuitively, obese PH patients maintain decreased mortality rates compared to non‐obese PH patients (Frank et al. 2020). Circulating hypotheses regarding the obesity paradox in PH include paradoxical right ventricular function preservation upon the onset of PH in obese patients, as well as oxidative stress and pro‐inflammatory cytokine reduction as a result of PH treatment (Yazdi et al. 2025). A recent study demonstrated an association between obesity and PH by determining elevated adipose tissue estrogen synthesis upregulated pulmonary CYP1A1 expression, a potential therapeutic target for obesity‐associated PH (Dignam, Sharma, et al. 2025). Further investigation is necessary to elucidate the underlying mechanisms behind the relationship between obesity and PH. Insulin resistance is commonly observed among patients, although its relationship with PH is not fully understood. Upregulation of obesity‐related inflammatory cytokines, such as TNF‐α and IL‐6, and activation of inflammatory pathways, such as NLRP3 inflammasome, constitute some of the molecular determinants that illustrate their relationship (Figure 1; Zanotto et al. 2023). Metabolic dysfunction, specifically insulin resistance, and its association with PH requires further exploration.

Retinal vascular changes have been characterized by increased retinal vascular tortuosity, as illustrated in Figure 1 (DuPont et al. 2022). These ocular manifestations potentially precede clinical detectability and therefore could be utilized as a non‐invasive disease detection method (DuPont et al. 2022). Underlying molecular mechanisms between retinal vasculature changes and PH remain largely unknown.

Bone marrow‐derived cells, such as hematopoietic stem cells (HSCs) and endothelial progenitor cells (EPCs), induce pulmonary vasculature alterations; however, it's unclear whether they have a causal effect or emerge as a result of the disease process (Figure 1). Some evidence suggests circulating dysfunctional HSCs and cytokine upregulation in the lung initiate PH pathogenesis, which implies HSC recruitment occurs in response to systemic inflammation (Yan et al. 2016). Other evidence suggests extracellular vesicles released from pulmonary hypertensive vasculature induce pathogenic EPC differentiation in the bone marrow, which in turn perpetuates disease phenotype (Aliotta et al. 2017). Together, these alterations throughout the body support the idea that PVD represents a systemic disorder, rather than simply just the heart and lungs. By understanding these broader physiological interactions, we can better understand the human body while simultaneously looking at more potential therapeutic targets of the heart and lung.

## 6 Future Directions and Clinical Implications

The recognition of pulmonary vascular disease as a disorder of integrated, multi‐organ communication has important implications for both research and clinical care. While current therapies primarily target pulmonary vascular tone and resistance, the evidence presented throughout this review suggests that disease progression is driven by coordinated, multidirectional signaling between the pulmonary vasculature, right ventricle, nervous system, immune system, and peripheral organs. As such, therapeutic strategies that focus solely on the pulmonary vasculature are unlikely to fully address the complexity and progression of disease.

Further research should prioritize a systems‐level approach that integrates hemodynamic data with molecular, inflammatory, metabolic, and extracellular vesicle‐based signaling pathways. Additionally, advances in technology, such as single‐cell sequencing, spatial transcriptomics, and circulating biomarker profiling, provide new and exciting opportunities to better define inter‐organ communication and possibly identify biologically distinct disease phenotypes. In parallel, data‐driven approaches, including the use of artificial intelligence and machine learning, offer the potential to integrate multimodal datasets into unified predictive frameworks, improving risk stratification, predicting right ventricular failure, and arguably most importantly, identifying novel therapeutic targets.

From a clinical perspective, these insights could support a broader framework for therapeutic development. In addition to already existing pulmonary vasodilators, interventions targeting neurohormonal dysregulation, pathologic inflammation, and extracellular vesicle‐mediated signaling may offer an additive benefit. Most importantly, future clinical trial design should consider pulmonary vascular disease as a multisystem disorder, incorporating endpoints that reflect right ventricular function, systemic inflammation, skeletal muscle performance, and functional capacity, rather than solely relying on pulmonary hemodynamics.

As advances in therapy continue to improve survival, additional considerations such as aging‐related changes in vascular stiffness, immune regulation, and right ventricular reserve will become increasingly relevant in shaping long‐term outcomes. These age‐related alterations may influence disease progression, therapeutic response, and the development of right ventricular failure, suggesting that future studies should consider age‐specific mechanisms and potentially stratified treatment approaches.

Together, these directions emphasize the need to move towards a more integrated model of pulmonary vascular disease, one that recognizes the dynamic and multidirectional communication between the lungs, right ventricle, and other organ systems. Targeting these interconnected pathways may ultimately lead to more precise and effective therapeutic strategies.

## 7 Conclusions

The interaction between the RV and pulmonary vasculature extends beyond the physical connection between the heart and lungs; rather, it encompasses neurohormonal, inflammatory, extracellular vesicles, and metabolic communication networks (Table 1). A deeper understanding of this axis may in the future result in the development of new therapeutic targets to improve the outcomes of pulmonary vascular disease. Although communication exists beyond the physical connection, hemodynamic coupling remains central to the long‐term prognosis; however, the pathways discussed above help us better understand the trajectory of the disease. Neurohormonal activation via RAAS and sympathetic reflex arcs originating in the lungs further amplifies pulmonary vasoconstriction and accelerates right ventricular remodeling. Furthermore, the natriuretic peptide system provides an important counter‐regulatory signal that links right ventricular stress to vascular protection. Inflammation further connects the heart and lungs, since cytokines, chemokines, and damage‐associated molecular patterns create parallel inflammatory niches that propagate remodeling in both the vascular wall and myocardium. Recently, extracellular vesicles have emerged as an additional pathway in which the heart and lungs communicate via the transportation of microRNAs, proteins, and metabolites between the heart and lung, which further propagates disease.

**TABLE 1:** Summary of inter‐organ communication in pulmonary vascular disease.

Organ/System	Key cells/Components	Current understanding	Future directions/Clinical implications
Pulmonary vasculature	Endothelial cells, smooth muscle cells	Central driver of increased PVR and remodeling due to the release of cytokines, EVs and vasoactive mediators (Ruopp and Cockrill 2022; Humbert et al. 2022)	Target endothelial dysfunction, EV signaling and vascular remodeling pathways
Right ventricle (RV)	Cardiomyocytes, fibroblasts	Responds to increased afterload with hypertrophy, ultimately leading to heart failure. Releases natriuretic peptides and DAMPs (Harrison et al. 2015; Veerdonk et al. 2011)	Improve RV specific therapies and enhance contractility and prevent maladaptive remodeling
Neurohormonal system	SNS, RAAS, NP system	Mediates bidirectional signaling between the heart and lung, contributing to vasoconstriction and remodeling (de Man et al. 2012; Vaillancourt et al. 2017; Kuwahara 2021)	Target autonomic imbalance, RAAS modulation and enhance NP signaling
Immune system	Macrophages, T cells, cytokines (IL‐6, TNF‐α, IL‐1β)	Drives inflammation in both lung and RV, promoting remodeling and dysfunction (Hu et al. 2020; Tomaszewski et al. 2021)	Develop targeted anti‐inflammatory therapies and immune modulation strategies
Extracellular vesicles	Exosomes, micro vesicles, miRNA	Mediate molecular communication between lung, heart and peripheral organs (Letsiou and Bauer 2018; Aliotta et al. 2016)	Use as biomarkers and develop EV‐based or targeted therapies
Central nervous system	Cortex, brainstem, autonomic circuits, spinal cord	Regulates sympathetic outflow in response to hypoxia and vascular stiffness, ultimately contributing to disease progression (Iturriaga et al. 2021; Vaillancourt et al. 2017)	Further explore neuromodulation therapies (PADN, vagal stimulation, etc.)
Skeletal muscle	Myocytes, mitochondria	Exhibits mitochondrial dysfunction and reduced oxygen utilization, which contributes to exercise intolerance (Riou et al. 2020)	Target metabolic dysfunction and improve functional capacity
Gastrointestinal system	Gut epithelium, microbiome	Altered absorption and microbiome contribute to systemic inflammation (Zanotto et al. 2023; Moutsoglou et al. 2023)	Investigate gut‐lung axis and microbiome‐targeted therapies
Liver	Hepatocytes, inflammatory signaling pathways	Contributes to systemic inflammation and endothelial dysfunction via cytokine signaling (Singh, Lawson, et al. 2025; Singh, Volpicelli, et al. 2025; González‐Teshima et al. 2025; Abdulkarim et al. 2023)	Target liver derived inflammatory pathways and angiocrine signaling
Kidney	Renal vasculature, RAAS activation	Cardiorenal interactions promote volume overload and vascular remodeling (Yuan et al. 2024; Zeder et al. 2024)	Address cardiorenal syndrome and optimize volume and RAAS control/input
Adipose/Metabolic system	Adipocytes, insulin signaling pathways	Links obesity, inflammation and insulin resistance to PH pathogenesis (Frank et al. 2020; Zanotto et al. 2023)	Explore metabolic therapies and obesity‐related mechanisms

*Note:* Key organ systems and their respective cellular components involved in the pathogenesis of pulmonary vascular disease are outlined, along with our current understanding of their respective roles in cardiopulmonary signaling and potential future research and therapeutic directions.

Taken together, these findings support a model in which the pulmonary vasculature and right ventricle function as an integrated biological unit. Future therapies will likely need to target not only mechanical load but also the molecular and cellular communication pathways that sustain disease. Understanding and modulating this heart–lung dialog may ultimately lead to more precise risk stratification and more effective treatments for pulmonary vascular disease.

## Author Contributions

A.A.V. and S.U. contributed to the conception and design of the overall review article. The writing, revising, editing and feedback was performed by all authors. Figures were created by J.M.A. and M.E. with feedback from the authors. Supervision was done by S.U.

## Funding

This work was supported by the National Institutes of Health (R01HL161038).

## Conflicts of Interest

The authors declare no conflicts of interest.

## Data Availability

Data sharing not applicable to this article as no datasets were generated or analysed during the current study.
